# Supplementary material for: Metabolome and proteome analyses reveal transcriptional misregulation in glycolysis of engineered E. coli
Source: Nat Commun. 2021 Aug 13;12:4929. doi: 10.1038/s41467-021-25142-0 (PMC8363753; doi:10.1038/s41467-021-25142-0)
Supplement: Supplementary file 1 — Supplementary Information [file 41467_2021_25142_MOESM1_ESM.pdf]

Supplementary Information

Metabolome and proteome analyses reveal transcriptional misregulation in glycolysis of engineered *E. coli*

Chun-Ying Wang, Martin Lempp, Niklas Farke, Stefano Donati, Timo Glatter, Hannes Link

List of Supplementary Information

• **Supplementary Figures 1–11**

• **Supplementary Tables 1–2**

Supplementary Figures

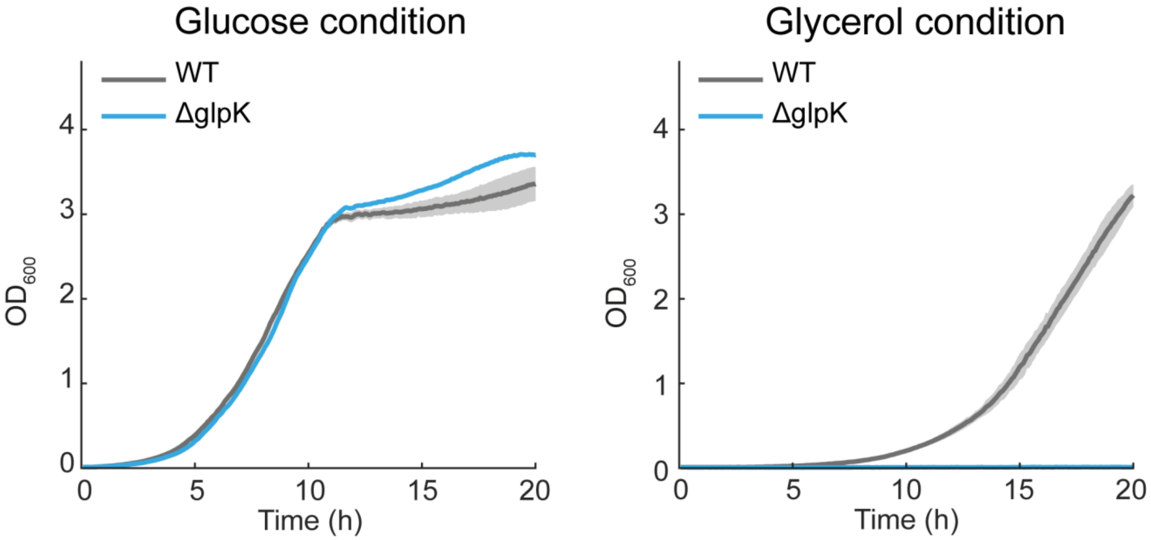

Supplementary Figure 1. Growth curves of *E. coli* wild-type and *E. coli* △*glpK*. The two strains were cultivated in 96-well plates with M9 medium supplemented with 0.5% glucose (left) or 0.5% glycerol (right). OD_{600} was measured in n = 2 plate reader cultures.

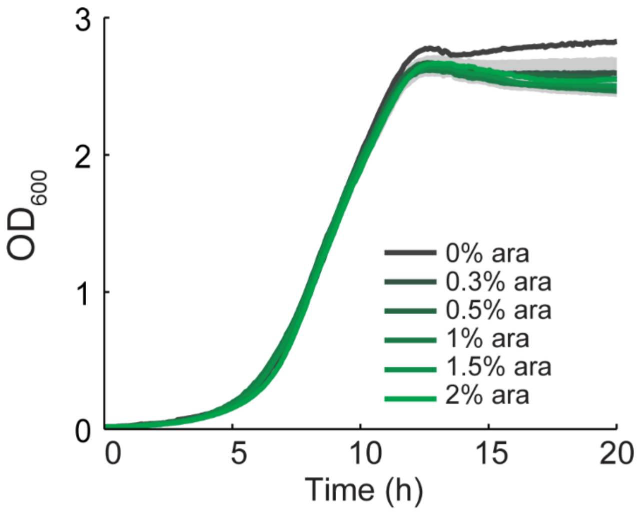

Supplementary Figure 2. Growth curves of GFP producing *E. coli*. GFP was expressed from the pBAD promoter in wild-type *E. coli* and the strain was cultivated in 96-well plates with varying arabinose concentrations. OD_{600} was measured in n = 2 plate reader cultures.

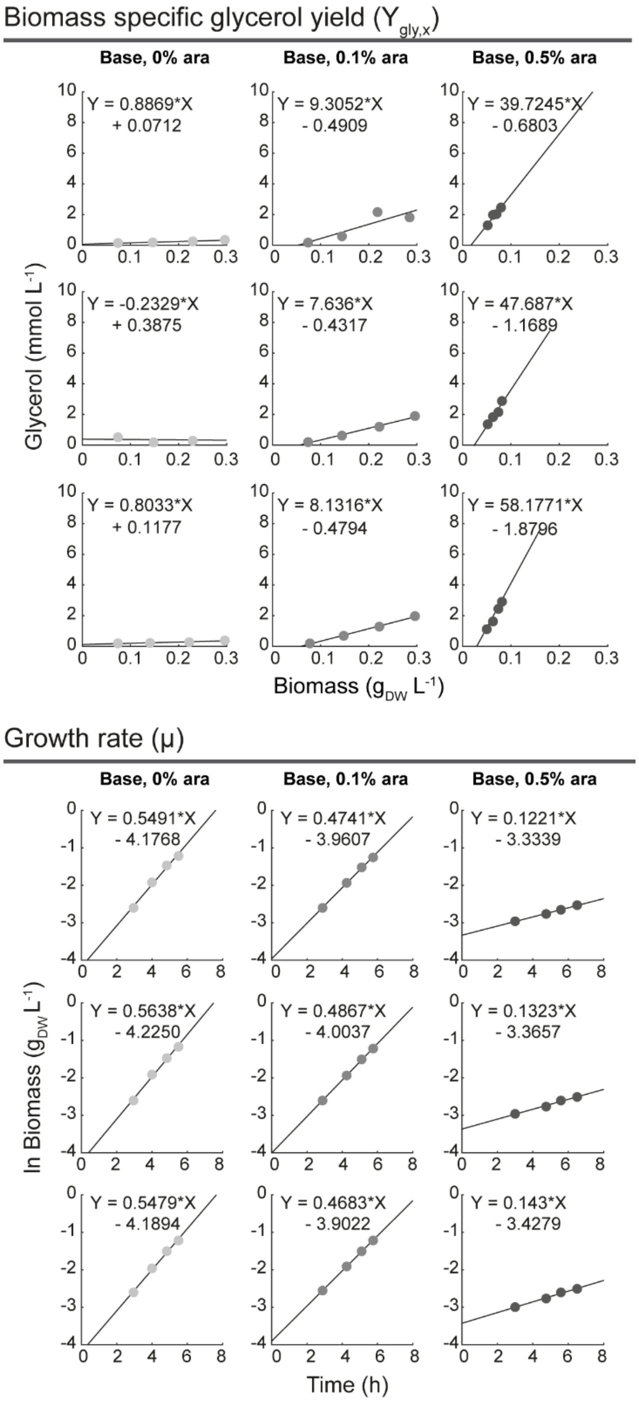

Supplementary Figure 3. Biomass specific glycerol yields and growth rates of the base strain. Biomass specific glycerol yield ($Y_{gly,x}$) of cultures with 0%, 0.1% and 0.5% ara was calculated by linear regression of glycerol concentration (mmol L^{-1}) and the biomass (g_{DW} L^{-1}) for 3 shake flask cultures per strain. Growth rates (µ) were calculated at the same time points by linear regression of *ln* biomass (g_{DW} L^{-1}) and the time (h). Specific glycerol production rates follow as $q_{gly} = Y_{gly,x} \bullet \mu$, and they are shown in Fig. 1e. When the slope of biomass specific glycerol yields was negative, the specific glycerol production was set to zero.

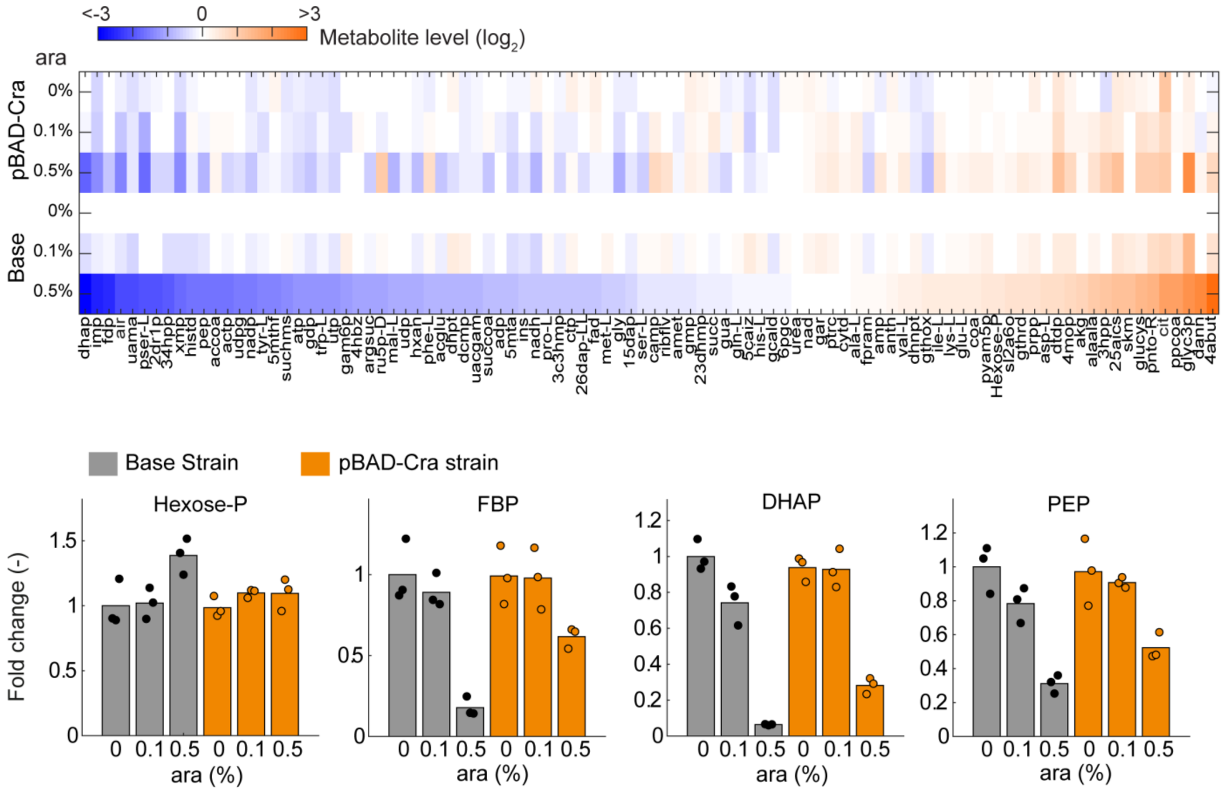

Supplementary Figure 4. Metabolome of base and pBAD-Cra strain. The heatmap shows the concentration of 96 intracellular metabolites in the base strain and the pBAD-Cra strain at 0%, 0.1% and 0.5% arabinose. Data is normalized to the 0% culture of the base strain. The bar plots show concentrations of hexose-phosphates (Hexose-P), fructose 1,6-bisphosphate (FBP), dihydroxyacetone phosphate (DHAP) and phosphoenolpyruvate (PEP) at 0%, 0.1% and 0.5% arabinose. Data are means from n = 3 independent shake flask cultures.

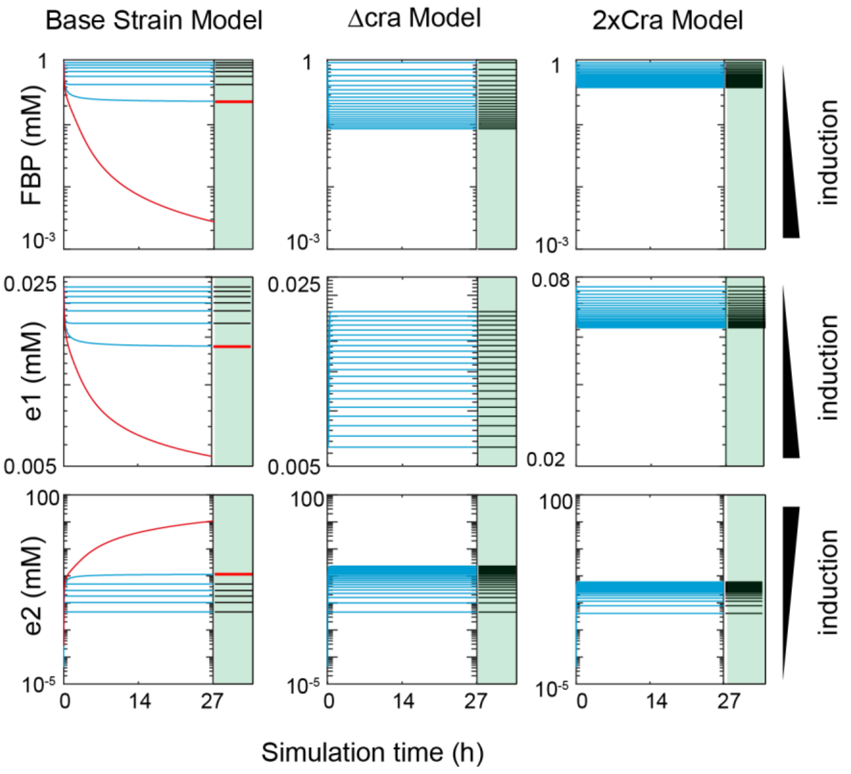

Supplementary Figure 5. Time-course simulation of the three models with an average parameter set and results with the parameter continuation method (green panel). Each of the three models (base strain model, 2xCra model, Δ*cra* model) was simulated with the average parameter set (median from intervals in Table 1). Blue lines are different induction levels from *ind* = 0 until 1 (for the 2xCra model, Δ*cra* model), or until the calculated bifurcation point (for the base strain model). The red line for the base model is the time-course of an induction level that is 1% stronger than at the bifurcation point. The panels with green background show the steady states calculated by the continuation method. Black lines are stable steady states and a red line indicates a bifurcation point.

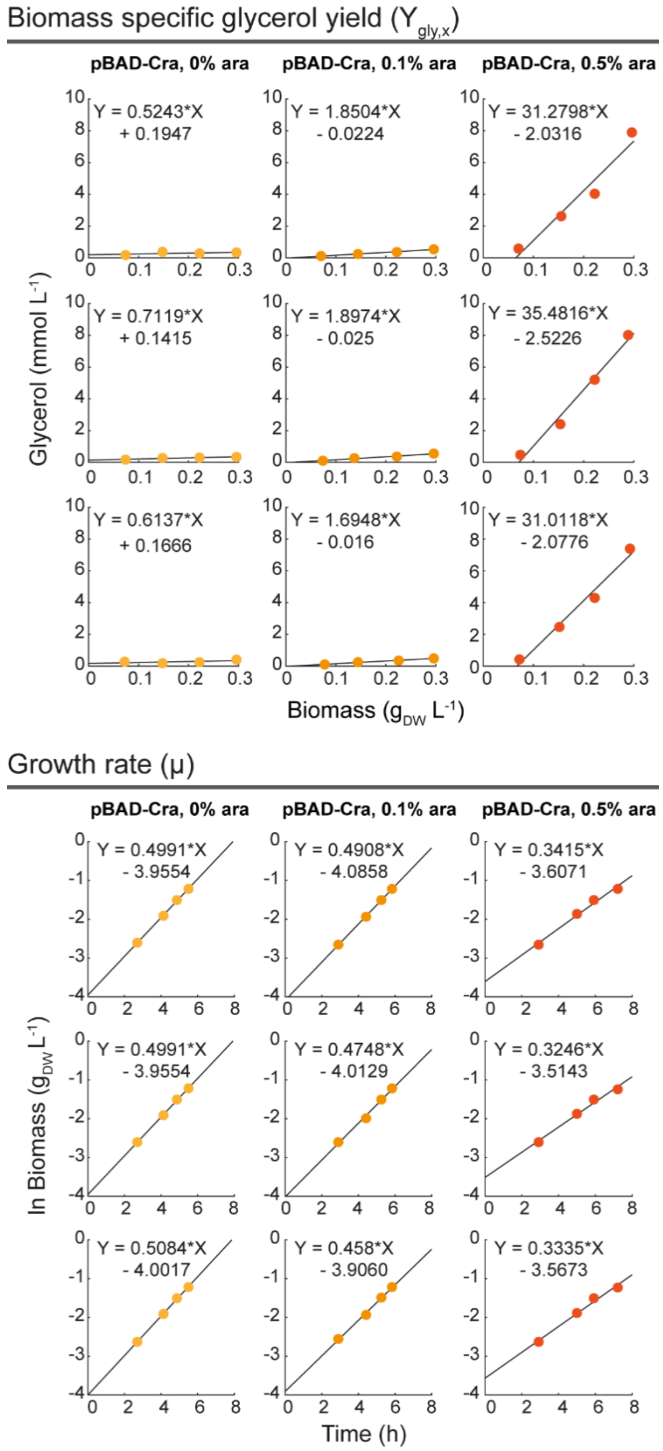

Supplementary Figure 6. Biomass specific glycerol yields and growth rates of the Cra-regulated pBAD strain. Same as **Supplementary Figure. 3** for the pBAD-Cra strain.

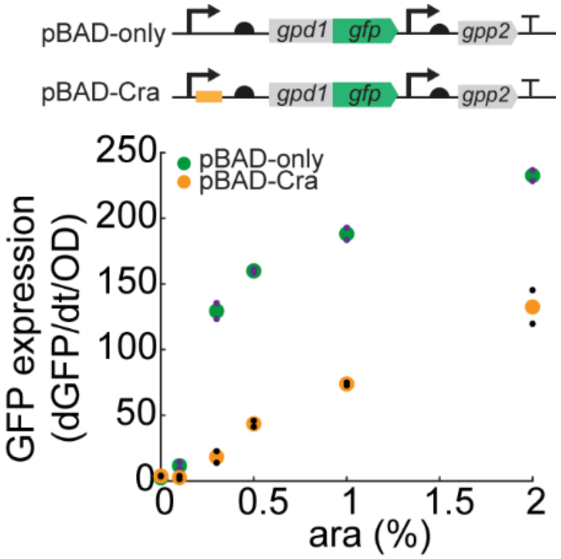

Supplementary Figure 7. Expression of a GPD1-GFP fusion protein in the base strain (green) and the pBAD-Cra strain (orange). GPD1-GFP was expressed from the pBAD promoter and the pBAD-Cra promoter. GFP fluorescence and OD_{600} were measured in n = 2 plate reader cultures, and promoter activity was calculated as dGFP/dt/OD by regression analysis between 7 and 9 h.

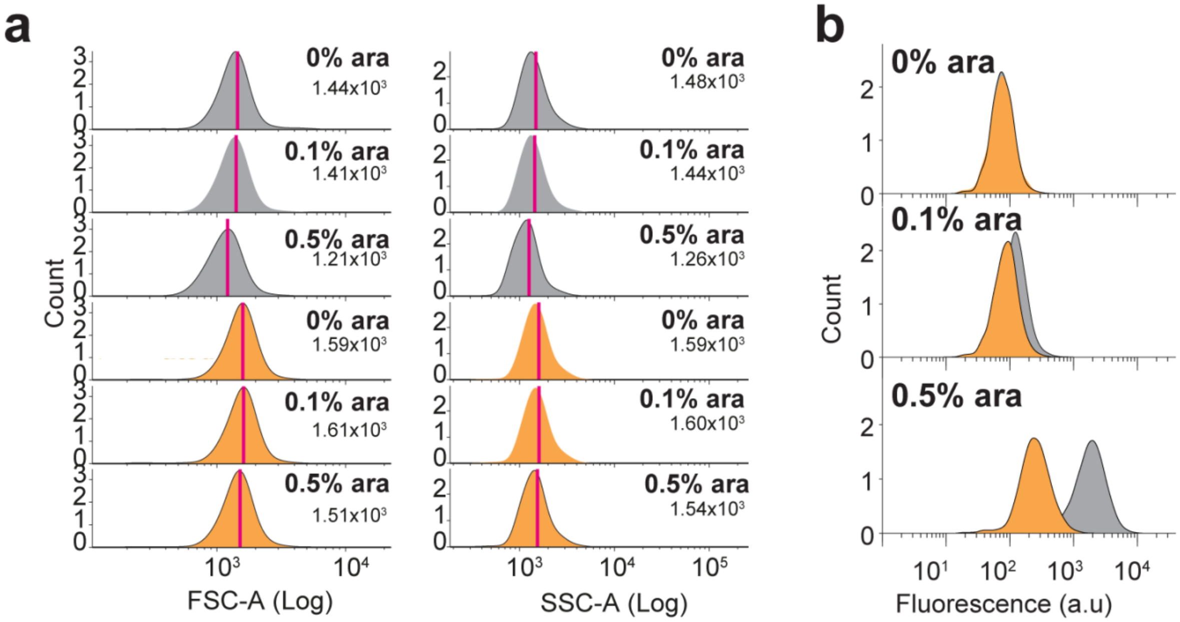

Supplementary Figure 8. Flow cytometry data of the GPD1-GFP fusion protein expressed in the base strain (grey) and the pBAD-Cra strain (orange). a, Forward (FSC-A) and side scatter (SSC-A) of the strains expressing GPD1-GFP from the pBAD promoter without (grey color) or with a Cra-binding site (orange color). The strains were cultured with 0%, 0.1% and 0.5% arabinose. The pink line is the mean of the distribution. **b,** Same as (a) for GFP fluorescence.

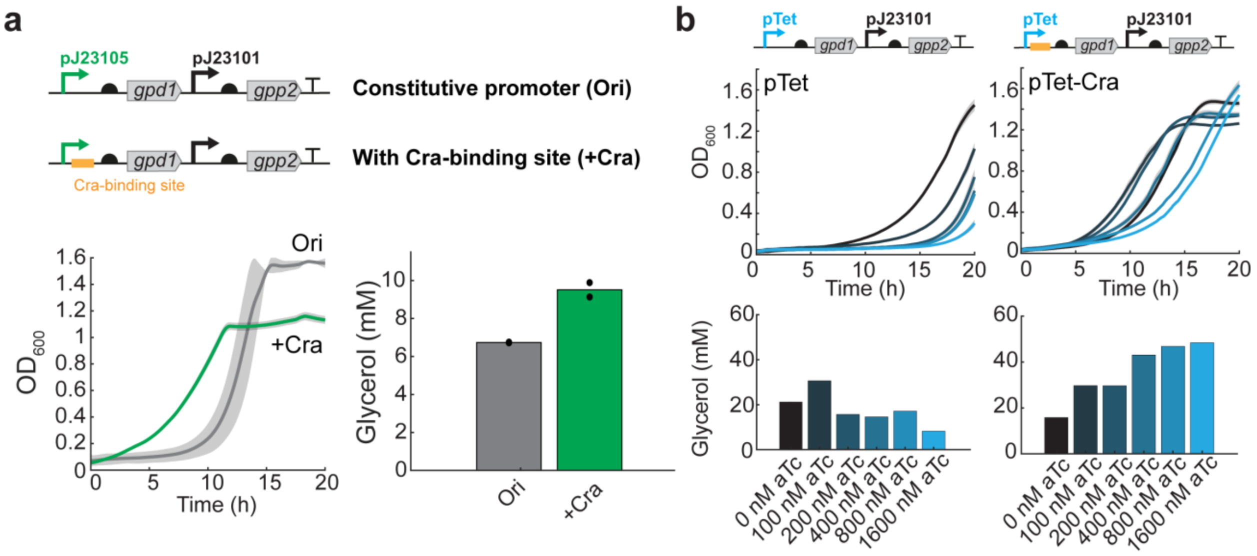

Supplementary Figure 9. Expression of the glycerol pathway with a constitutive and a pTet promoter. **a,** GPD1 was expressed with a constitutive promoter-pJ23105 (ori, original) and the same promoter with a Cra binding site (+Cra). The resulting strains were cultured in 96 well plates. Growth was measured in a plate reader and glycerol concentration was measured after 24 h. Growth curves and bar plots show means of n = 2 plate reader cultures. **b,** GPD1 was expressed with a pTet promoter and the same promoter with a Cra binding site (+Cra). The pTet promoter is an aTc inducible promoter and different levels of aTc were tested (0, 100, 200, 400, 800 and 1600 nM). n = 2 for growth curves and n = 1 for glycerol.

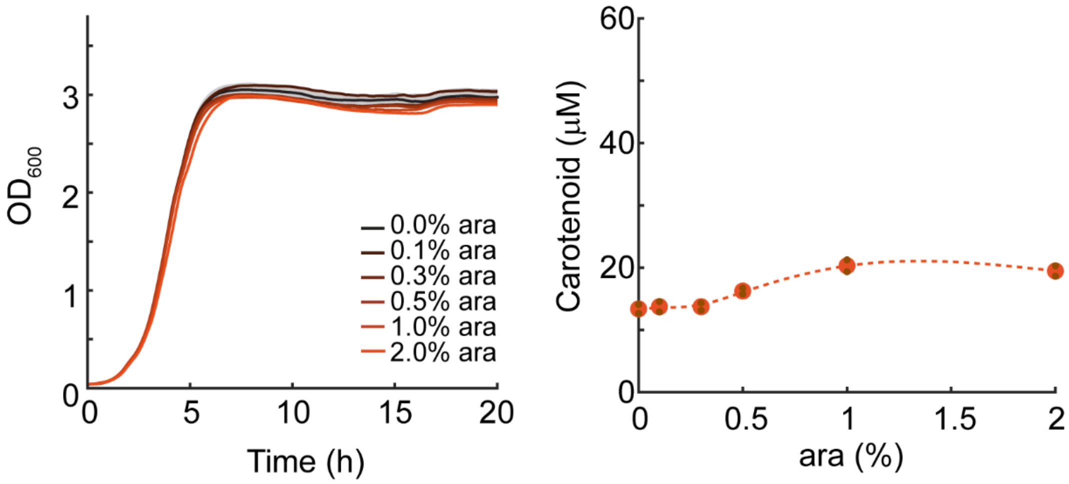

Supplementary Figure 10. Expression of the pCarotenoid plasmid in *E. coli* BW25113. Growth curves and carotenoid levels were measured in 96 well plate cultures (n = 2). The total carotenoid content was measured after 24 h. Growth curves and dots show means of n = 2 cultures, and grey shaded areas show the difference between two cultures.

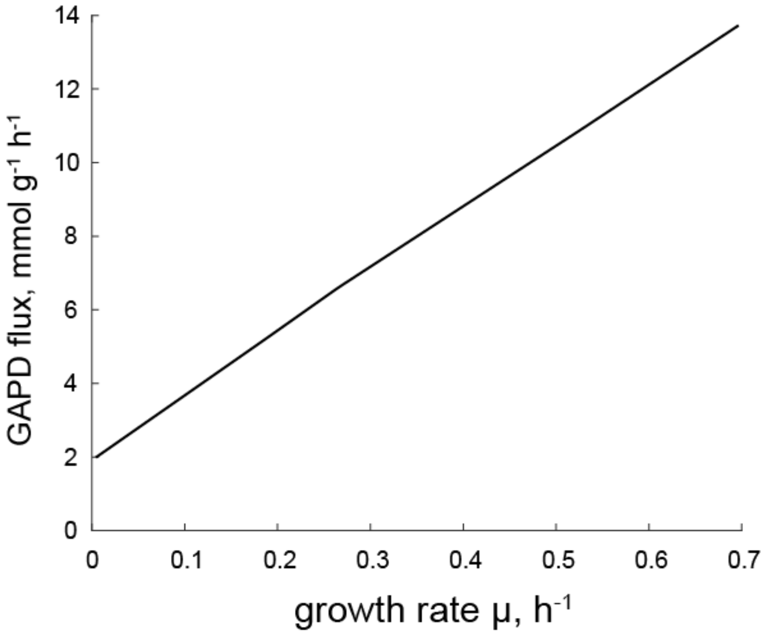

Supplementary Figure 11. Theoretical relationship between lower glycolytic flux and growth rate. The reaction rates of GAPD in lower glycolysis and growth rates were calculated with flux balance analysis and the genome-scale model *i*ML1515.

Supplementary Tables

Supplementary Table S1. Strains, plasmids and oligonucleotides used in this study.

Strains	Note	Reference / source
Escherichia. coli MegaX DH10B T1R	Cat#C640003, used during cloning	Invitrogen, Thermo Fischer Scientific
E. coli DH5α	Cat#18265017, used during cloning	Invitrogen, Thermo Fischer Scientific
E. coli MG1655	wild-type *E. coli* K-12: F^-, $lambda^-$, rph^{-1}	DSMZ No. 18039
E. coli BW25113	Wild-type *E. coli* K-12: $lacI^+$, Ara^- and Rha^-	Keio collection[1]
E. coli MG1655 Δ*glpK*	*E. coli* MG1655 strain: Δ*glpK* strain	This study
E. coli MG1655 Δ*cra*	*E. coli* MG1655 strain: Δ*cra* strain	This study
E. coli MG1655 Δ*glpK* Δ*cra*	*E. coli* MG1655 strain: Δ*glpK* and Δ*cra*	This study
pBAD (GFP)	*E. coli* MG1655 strain with pBAD-GFP plasmid	This study
pBAD-Cra (GFP)	*E. coli* MG1655 strain with pBAD-Cra-GFP plasmid	This study
Base strain	*E. coli* MG1655 Δ*glpK* strain with pBAD plasmid	This study
Cra-regulated strain	*E. coli* MG1655 Δ*glpK* strain with pBAD-Cra plasmid	This study
Base strain (carotenoid)	*E. coli* BW25113 strain with both pController and pCarotenoid plasmids	This study
Cra-regulated (carotenoid)	*E. coli* BW25113 strain with both pController-Cra and pCarotenoid plasmids	This study
**Plasmids**	**Note**	**Reference / source**
pKDsgRNA-ack	Template for cloning sgRNA, sgRNA under the control of constitutive promoter P_{J23119}, Spectinomycin, pSC101	Addgene plasmid #62654
pCas9cr4	Cas9 plasmid for no-SCAR genome editing, Chloramphenicol, p15A	Addgene plasmid #62655
pKDsgRNA-p15	Curation of the Cas9 plasmid, Spectinomycin, pSC101	Addgene plasmid #62656
pKDsgRNA-glpK	Expression of sgRNA targeting *glpK*, Spectinomycin, pSC101	This study
pKDsgRNA-Cra	Expression of sgRNA targeting *cra*, Spectinomycin, pSC101	This study
pBAD-GFP	GFP (BBa_E0040) under control of P_{BAD} promoter, p15A, Kanamycin, pSB3K3	Lin *et al.*[31]
pBAD-Cra-GFP	Plasmid pBAD-GFP with Cra-binding site	This study
pBAD	Arabinose inducible pBAD promoter for *gpd1* and the constitutive promoter P_{J23101} for *gpp2*, p15A, Kanamycin, pSB3K3	This study
pBAD-Cra	Same as pBAD plasmid but inserted Cra-binding site directly after pBAD promoter, p15A, Kanamycin, pSB3K3	This study
pBAD + 1Cra	Modified pBAD plasmid by adding additional 1 Cra consensus sequence after rrnB terminator, p15A, Kanamycin, pSB3K3	This study
pBAD-Cra + 1Cra	Modified pBAD-Cra plasmid by adding additional 1 Cra consensus sequence after rrnB terminator, p15A, Kanamycin, pSB3K3	This study
pBAD + 2Cra	Modified pBAD plasmid by adding additional 2 Cra consensus sequences after rrnB terminator, p15A, Kanamycin, pSB3K3	This study
pBAD-Cra + 2Cra	Modified pBAD-Cra plasmid by adding additional 2 Cra consensus sequences after rrnB terminator, p15A, Kanamycin, pSB3K3	This study
pController	Arabinose inducible pBAD promoter for *dxs* and *dxr*, p15A, Kanamycin, pSB3K3	This study
pController-Cra	Inserted Cra-binding site after pBAD promoter for *dxs* and *dxr*, p15A, Kanamycin, pSB3K3	This study
pCarotenoid	Native constitutive promoter of *Pantoea ananatis* drives carotenoid synthetic gene of *crtB*, *crtI*, *crtY*, *idi*, *crtE* from *P. ananatis* and also *dxs* from *E. coli* for supplemental precursors of EMP pathway, pMB1, Ampicillin	Professor Dr. Victor Sourjik, MPI, Marburg

Weaker-pBAD	Same as pBAD plasmid but with modified -10 and -30 region of pBAD promoter, p15A, Kanamycin, pSB3K3	This study

Supplementary Table S2. Oligonucleotides used in this study

Oligonucleotides	Sequence (5' to 3')	Use
glpk-sgRNA-F	CGTGATCCATTACGACCGCGgttttagagctagaaatagcaag	Amplification from pKDsgRNA-ack plasmid to obtain backbone fragments for construction of pKDsgRNA-glpK
SCAR-sgRNA-R	gtgctcagtatctctatcactga	
Knockout-glpk	CTACGGGACAATTAAACATGACTGAAAAAAAATATATCGTTGCGCTCGACCAGGGCACCACCAGCTCGGTTAAACGCGCGATGGCGTGGGAAGAACACGACGAAtaaTGTAAATGCCGAATG	Template sequence for deletion of *glpK* to create *E. coli* MG1655 Δ*glpK*
Cra-sgRNA-F	ACTGGATGAAATCGCTCGGCgttttagagctagaaatagcaag	Forward primer used with SCAR-sgRNA-R for construction of pKDsgRNA-Cra
Knockout-Cra	GATCTCAATGCGCAATTTACAGCCCAACATGTCACGTTGGCCGCGCCAGGTGAATTTCCCTCTGGCGCGTAGAGTACGGGACTGGACATC	Template sequence for deletion of *cra* to create *E. coli* MG1655 Δ*cra*
pBAD-F	AATTCTCATGTTTGACAGcttcctcgctcactgactcgc	Amplification from pBAD-GFP plasmid to obtain linear pBAD promoter fragments
pBAD-R	cagcagcagacatctagtaTTTCTCCTCTTTCTCTAGTAGCTAGCC	
GPD1-F	AGGAGAAAtactagatgTCTGCTGCTGCTGATAGATTAAACTTAAC	Amplification from yeast genomic DNA to obtain linear *gpd1* fragments
GPD1-R	GTCAATccccatataactaCAATCATGTCCGGCAGGTTCTTC	
GPP2-F	ACATGATTGtagttatatggggATTGACTACTAAACCTCTATCTTTGAAAGTTAACGC	Amplification from yeast genomic DNA to obtain linear *gpp2* fragments
GPP2-R	cacactaccatcttaCCATTTCAACAGATCGTCCTTAGC	
Gly-B-F	gatgGGATTGACTACTAAACCTCTATCTTTG	Amplification from pBAD-GFP to obtain backbone fragments
Gly-B-R	GTAGATCTAATTCTTCAATCATGTCCGGCAGGTTC	
J01B32	CCGGACATGATTGAAGAATTAGATCTACATGAAGATtagGCATGCGAGCTCCATATGACTAGTtttacagctagctcagtcctaggtattatgctagcTACTAGAGtcacacaggaaagtactagatgGGATTGACTACTAAACCTCTATCTTTG	The J23101 promoter for *gpp2*. Combination J01B32 with above all linear fragments by CPEC to obtain pBAD plasmid
Cra(-10)-F	CTCTAGTAGACTGAAACGCTTCAGCTGCTAGCCCAAAAAAACGGTATGG	Amplification from pBAD plasmid to obtain backbone fragments
Cra(-10)-R	AAAGAGGAGAAAtactagatgTCTGCTGC	
Template+Cra	AGCTGAAGCGTTTCAGTCTACTAGAGAAAGAGGAGAAAtactagatgTCTGCTGC	Combination Template+Cra with above linear fragments by Gibson assembly to obtain pBAD-Cra plasmid
Backbone-F	CTCGAGTCCCGTCAAGTCAG	Amplification from pBAD plasmid or pBAD-Cra plasmid as a backbone sequence for adding additional Cra-binding site after rrnB terminator sequence.
Backbone-R	ctgcctcggtgagttttctc	
Cra_1Temp	gctcactcaaaggcggtaatAGCTGAAGCGTTTCAGTCctcgagtcccgtcaagtcag	The template sequence for adding 1 additional Cra-binding site
Cra_2Temp	gctcactcaaaggcggtaatAGCTGAAGCGTTTCAGTCGCGATCTTGTCTTTAACCCTAAGCCAGGTTGGCGCTTTTTTTCTAGCTGAAGCGTTTCAGTCctcgagtcccgtcaagtcag	The template sequence for adding 2 additional Cra-binding sites
pBAD-Bdxs-F	gTACTAGAGccaggcatcaaataaaacgaaag	Amplification from pBAD plasmid to obtain linear backbone fragments. Combination linear *dxs* and *dxr* fragments by Gibson assembly to obtain pController.
pBAD-Bdxs-R	ctagtaTTTCTCCTCTTTGGTACCGCTAGCCCAAAAAAACGGTATGGAGAAAC	
pBAD-Cra-Bdxs-R	ctagtaTTTCTCCTCTTTGGTACCGACTGAAAC	With pBAD-Bdxs-F primer to amplify from pBAD-Cra plasmid to obtain linear backbone fragments. Combination linear *dxs* and *dxr* fragments by Gibson assembly to obtain pController-Cra.
dxs-F	GGTACCAAAGAGGAGAAAtactagatgAGTTTTGATATTGCCAAATACCCGAC	Amplification from *E. coli* MG1655 genomic DNA to obtain linear *dxs* fragments
dxs-R	ctagtaTTTCTCCTCTTTCATATGttaTGCCAGCCAGGCCTTGATTTTG	

dxr-F	cataaCATATGAAAGAGGAGAAAtactagatgAAGCAACTCACCATTCTGGGCTC	Amplification from E. coli MG1655 genomic DNA to obtain linear dxr fragments
dxr-R	tttgatgcctggCTCTAGTActgcaGtcaGCTTGCGAGACGCATCA	
Weaker-pBAD -F	ctgtattagtactgttggTCCATACCGTTTTTTTGGGCTAGCTAC	Amplification from pBAD plasmid to obtain linear fragments. Using inverse PCR to get plasmid 73X.
Weaker-pBAD X-R	ctgtattagtactgttggTCCATACCGTTTTTTTGGGCTAGCTAC	
VF2	TGCCACCTGACGTCTAAGA	For sequencing of promoter region
VR	ATTACCGCCTTTGAGTGAGC	For sequencing of promoter region
