# Supplementary material for: Metabolome and proteome analyses reveal transcriptional misregulation in glycolysis of engineered E. coli
Source: Nat Commun. 2021 Aug 13;12:4929. doi: 10.1038/s41467-021-25142-0 (PMC8363753; doi:10.1038/s41467-021-25142-0)
Supplement: Supplementary file 2 — Reporting Summary [file 41467_2021_25142_MOESM2_ESM.pdf]

nature research

Corresponding author(s): Hannes Link

Last updated by author(s): Jul 21, 2021

# Reporting Summary

Nature Research wishes to improve the reproducibility of the work that we publish. This form provides structure for consistency and transparency in reporting. For further information on Nature Research policies, see our Editorial Policies and the Editorial Policy Checklist.

## Statistics

For all statistical analyses, confirm that the following items are present in the figure legend, table legend, main text, or Methods section.

| n/a | Confirmed | |
|---|---|---|
| ☐ | ☒ | The exact sample size ($n$) for each experimental group/condition, given as a discrete number and unit of measurement |
| ☐ | ☒ | A statement on whether measurements were taken from distinct samples or whether the same sample was measured repeatedly |
| ☒ | ☐ | The statistical test(s) used AND whether they are one- or two-sided<br>*Only common tests should be described solely by name; describe more complex techniques in the Methods section.* |
| ☒ | ☐ | A description of all covariates tested |
| ☒ | ☐ | A description of any assumptions or corrections, such as tests of normality and adjustment for multiple comparisons |
| ☐ | ☒ | A full description of the statistical parameters including central tendency (e.g. means) or other basic estimates (e.g. regression coefficient) AND variation (e.g. standard deviation) or associated estimates of uncertainty (e.g. confidence intervals) |
| ☒ | ☐ | For null hypothesis testing, the test statistic (e.g. $F$, $t$, $r$) with confidence intervals, effect sizes, degrees of freedom and $P$ value noted<br>*Give P values as exact values whenever suitable.* |
| ☒ | ☐ | For Bayesian analysis, information on the choice of priors and Markov chain Monte Carlo settings |
| ☒ | ☐ | For hierarchical and complex designs, identification of the appropriate level for tests and full reporting of outcomes |
| ☒ | ☐ | Estimates of effect sizes (e.g. Cohen's $d$, Pearson's $r$), indicating how they were calculated |

*Our web collection on statistics for biologists contains articles on many of the points above.*

## Software and code

Policy information about availability of computer code

| | |
|---|---|
| Data collection | Progenesis QIP (Version 2.0)<br>MASCOT (v2.5, Matrix Science)<br>Agilent MassHunter LC/MS Data Acquisition (Version B.07.01) |
| Data analysis | Matlab (2018b) and Matlab (version 1.3.0.0)<br>SafeQuant |

For manuscripts utilizing custom algorithms or software that are central to the research but not yet described in published literature, software must be made available to editors and reviewers. We strongly encourage code deposition in a community repository (e.g. GitHub). See the Nature Research guidelines for submitting code & software for further information.

## Data

Policy information about availability of data

All manuscripts must include a data availability statement. This statement should provide the following information, where applicable:

- Accession codes, unique identifiers, or web links for publicly available datasets
- A list of figures that have associated raw data
- A description of any restrictions on data availability

Proteome raw data are deposited at the PRIDE database under the accession numbers: PXD022965 (https://www.ebi.ac.uk/pride/archive/projects/PXD022965). Metabolome raw data and flow cytometry data are deposited at the Open Research Data Repository of the Max Planck Society (Edmond) at: https://edmond.mpdl.mpg.de/imeji/collection/67E9ibZDJRkXQh1s. Source data are provided with this paper as source_data.xlsx.

April 2020

# Field-specific reporting

Please select the one below that is the best fit for your research. If you are not sure, read the appropriate sections before making your selection.

- [x] Life sciences
- [ ] Behavioural & social sciences
- [ ] Ecological, evolutionary & environmental sciences

For a reference copy of the document with all sections, see nature.com/documents/nr-reporting-summary-flat.pdf

# Life sciences study design

All studies must disclose on these points even when the disclosure is negative.

| | |
|---|---|
| Sample size | No sample size calculation was perfomed. Sample size was:<br>i) For continuous growth and fluorescence measurements in plate readers, sample size was n= 2, because multiple time points were measured.<br>ii) For growth, metabolome and proteome data in Figure 2, sample size was n= 3, because one time point was measured.<br>iv) For growth, glycerol, metabolome and proteome data in Figure 5, sample size was n= 2, because mulitple time points were measured.<br>ii) For glycerol and carotenoid measurements in plate readers, the sample size per plate reader run was n= 2, because many conditions (several promoters and induction levels) were measured. |
| Data exclusions | No data exclusion |
| Replication | Plate reader experiments with the base strain, the deltaCra strain and the pBAD-Cra were replicated multiple times at different days, and all attempts were succesfull. |
| Randomization | No randomization.  Covariates were controlled for by internal standards in case of metabolome measurements and running controls when applicable. |
| Blinding | No blinding, because sampling and LCMS analyses were performed and confirmed by independent researchers in our labs and the proteomics facility. |

# Reporting for specific materials, systems and methods

We require information from authors about some types of materials, experimental systems and methods used in many studies. Here, indicate whether each material, system or method listed is relevant to your study. If you are not sure if a list item applies to your research, read the appropriate section before selecting a response.

## Materials & experimental systems

| n/a | Involved in the study | |
|---|---|---|
| ☒ | ☐ | Antibodies |
| ☒ | ☐ | Eukaryotic cell lines |
| ☒ | ☐ | Palaeontology and archaeology |
| ☒ | ☐ | Animals and other organisms |
| ☒ | ☐ | Human research participants |
| ☒ | ☐ | Clinical data |
| ☒ | ☐ | Dual use research of concern |

## Methods

| n/a | Involved in the study | |
|---|---|---|
| ☒ | ☐ | ChIP-seq |
| ☒ | ☐ | Flow cytometry |
| ☒ | ☐ | MRI-based neuroimaging |
